# Evaluating Social Support and T2D Risk Factors Among Members of Rural-Dwelling Grandparent-Headed Households

**DOI:** 10.13023/jah.0402.06

**Published:** 2022-07-01

**Authors:** Brittany L. Smalls, Abebola Adegboyega, Kelly NB Palmer, Jennifer Hatcher

**Affiliations:** University of Kentucky, brittany.smalls@uky.edu; University of Kentucky; University of Arizona; University of Arizona

**Keywords:** Appalachia, rural health, grandparent-headed households, family, type 2 diabetes

## Abstract

**Purpose:**

This study examines the associations of social support and type 2 diabetes (T2D) risk factors among members of rural-dwelling, grandparent-headed households (GHH).

**Methods:**

Prospective data were collected from rural-dwelling members of GHH with no known diagnosis of T2D. Data collected on family characteristics, T2D clinical risk factors, and social support were assessed.

**Results:**

Sixty-six grandparents and 72 grandchildren participated in the study. The average age and HbA1Cs were 59.4 years and 6.2% ± 1.4 for grandparents and 11.8 years and 4.9% ± 0.6 for grandchildren. Most grandparents were found to have prediabetes or undiagnosed diabetes. The number of people living in GHHs was associated with grandparents’ triglycerides, HDL, and BMI. Average social support scores among grandparents suggested moderately high perceived social support (79 ± 3.4). For grandchildren, social support from grandparents was associated with diastolic blood pressure and HbA1C, whereas support from teachers, classmates, and close friends was associated with HbA1C and BMI in grandchildren.

**Implications:**

This study shows that grandparent caregivers are at an increased risk for T2D. Perceived social support between grandparents and grandchildren influences T2D risk factors. However, social support provided by peers, teachers, and close friends is also associated with T2D risk factors in grandchildren. These findings support the use of family-based diabetes prevention programming, peer support, and school settings as mechanisms for interventions to reduce T2D in adolescents, particularly those within GHHs.

## INTRODUCTION

Over 34 million persons in the U.S. live with type 2 diabetes (T2D), making it a major public health threat.^1^ Current estimates by the Centers for Disease Control and Prevention (CDC) project that by 2050 half of the U.S. adult population will have T2D, and one in three children in the U.S. will develop T2D in their lifetime.^2^ Residents of rural Appalachia suffer rates of T2D that are among the highest and most rapidly increasing in the country.^3^ Since 2000, the rates of T2D among adults in Kentucky have doubled to 12.9%. In Appalachia, rates are even higher, with 17% of adults having been diagnosed with T2D.^3^ Current diabetes prevention interventions primarily focus on individual-level modifiable physiological risk factors (e.g., weight loss, increasing physical activity, and improving sleep duration and quality). While these interventions have been effective in urban settings, dissemination in rural settings has been limited.

Of great concern is the expected four-fold increase in type 2 diabetes prevalence among youth.^4^ A particularly vulnerable population are children under the custodial care (formal or informal) of their grandparent(s). With almost 6 million children (aged <17 years) living with a grandparent, grandparent-headed households (GHH) are one of the fastest-growing family constellations in the U.S.^4^ One subset of GHH—skipped-generation families—are most common in rural areas.^5^ In Kentucky, more than 100,000 grandchildren are raised by their grandparents, and almost 60,000 grandparents are responsible for the care of their grandchildren. Furthermore, in the Appalachian counties of Kentucky, more than half of these GHH have no parent present.^6^

GHH experience many socioeconomic factors (e.g., education level, employment status, income level, family size, and social class) associated with increased risk for T2D, such as poor nutrition and sedentary behavior. The pervasiveness of poverty in some rural areas—alongside the overall difficulty in accessing health care, the scarcity of health and social services, and the physical and social environment—yield unique challenges for Appalachian communities. These challenges include being under-or uninsured, food insecurity, and limited access to resources. These characteristics are known determinants of health and health behaviors.

While GHH are likely to experience risk factors that predispose their members to the development of T2D, social support that is provided by relationships with family members, friends, and members of one’s social network can serve as a coping mechanism, mediator, or moderator to poor health outcomes. Social support from family, friends, or other significant relationships could modify the stressors experienced by members of GHH, decreasing vulnerability to health threats such as T2D.^7^ More is known about the effects of social support on diabetes self-management than about social support’s association with risk factors for T2D.

Efforts to address the inherent health challenges GHH face have primarily focused on the grandparents and those residing in urban settings. In this context, interventions to improve grandparents’ mental and physical health have demonstrated, on average, moderate positive effects at best.^7^ One study implemented a multimodal, home-based intervention to improve physical health status (e.g., glucose, blood pressure, lipids, physical functioning) that included shared goal setting, health assessments, and medical referrals and yielded significant changes in several outcomes.^8^ Other studies aimed to increase social support and resource acquisition for grandparents through group-based interventions and case management provided by social workers.^9^–^11^ Very few studies have actively engaged grandchildren in the intervention, and furthermore, there is little to no reported data on outcomes related to T2D risk factors.

Although many factors are understood to impact T2D risk, there is a paucity of information on socioecological factors that influence the risk for T2D among GHH. It is recognized that development of effective health promotion strategies for rural-dwelling GHH requires an understanding of the factors that influence their risk behaviors and that may be amenable to intervention. The objective of this study was to examine the associations of social support and T2D risk factors among members of rural-dwelling GHH.

## METHODS

### Participants

The target population for this study consisted of families residing in Appalachian Kentucky in which grandparents were the primary caregiver for their grandchild(ren). Of note, study participants lived in a county with a Rural–Urban Commuting Area (RUCA) code of 10, indicating a rural, geographically isolated area.

### Inclusion and Exclusion Criteria

Grandparents were eligible if (1) they were raising one or more grandchildren aged 10 to 14 years and (2) one or more family members was at risk for T2D, operationalized as being overweight (BMI >25 for adults; children >85^th^ percentile in weight for age and gender). In addition, participants self-reported one or more of the following risk factors: physical inactivity; first-degree relative with diabetes; hypertension; HDL cholesterol level <35 mg and/or triglyceride level >250 mg/dL; HGA1c >5.7; other clinical condition associated with insulin resistance (e.g., severe obesity, acanthosis nigricans); or history of cardiovascular disease. Individuals with type 1 diabetes were excluded from the study.

### Recruitment

A convenience sample of grandparents were recruited from Letcher and Perry Counties, two Southeastern Appalachian counties that are part of the Kentucky River District. Individuals were recruited for the study by (1) advertising in local newspapers and gazettes; (2) advertising at local churches, community centers, agricultural extension offices, senior centers, local business organizations, departments, and public fairs of all types; and (3) word of mouth. Participants received a $50 gift card for participating in the study.

### Study Design

This was a cross-sectional study design where data were collected regarding family structure, personal factors, clinical outcomes relevant to risk of T2D, and self-reported social support. All clinical outcomes data were collected during the study visit after participants provided written consent. All study procedures were approved by the institutional review board and approval was obtained via the Office of Research Integrity at the University of Kentucky (protocol #14-0311-PIH).

### Outcome Measures

#### Social Support

Social support was measured using the Medical Outcomes Study Social Support Scale (MOS-SSS)^12^ for grandparents. The MOS-SSS includes 19 questions with a five-point Likert scale for responses. Overall scores range from 0 to 100. The psychometric properties of the scale have good item variability, have good construct validity, and a Cronbach’s alpha of 0.97. In grandchildren, social support was measured using the Child and Adolescent Social Support Scale (CASSS).^13^ The CASSS includes 40 questions with two main themes: frequency of social support and the perceived importance of the support received. For each theme there are four subscales based on the individual providing social support: grandparent, teacher, classmate, and close friend. Under the theme of frequency of social support for each subscale, the score ranges from 12 to 72, where a higher score indicates more support. For the perceived importance theme, each subscale score ranges from 12 to 36, where a higher score indicates a higher perceived importance of receiving social support from that individual. Overall, the CASSS has a Cronbach’s alpha ranging from 0.94 to 0.95, based on grade level.

#### Body mass index (BMI)

Research personnel performed BMI measurement using standardized procedures. BMI was calculated from height and weight measured with a professional-grade stadiometer and a professional-grade digital body weight scale. A BMI of 26 or greater was considered overweight.

#### Blood pressure

Following at least 5 minutes of rest, participants had their blood pressure measured using American Heart Association standards by trained research personnel.^14^ A systolic blood pressure greater than 130mmHg or diastolic blood pressure greater than 80mmHg was considered elevated.

#### Lipids

For each participant, a full fasting lipid profile (i.e., total cholesterol, high- and low-density lipoprotein [HDL and LDL] and triglycerides) was analyzed using the Cholestech LDX^™^ System. Accuracy and reproducibility of the Cholestech LDX^™^ has been certified by the Cholesterol Reference Method Laboratory Network, demonstrating that this point-of-care lipid profile method is comparable to centralized laboratory testing.^15^ For the purposes of this study, lipids were considered problematic if LDL was greater than 130 mg/dL, HDL was less than 60 mg/dL, total cholesterol was greater than 200 mg/dL, and/or triglycerides were greater than 150 mg/dL.^16^

#### Hemoglobin A1c (HbA1c)

HbA1c was measured using the Bayer A1CNow+ Point of Care A1C (Bayer Healthcare) monitor and disposable test cartridge using a finger-stick whole-blood sample. Participants in this study who had an HbA1c between 5.7% (39 mmol/mol) and 6.4% (46 mmol/mol) were determined to be prediabetic, and those with an HbA1c ≥ 6.5% (48 mmol/mol) were considered to have T2D.^17^

### Statistical Analysis

Mean, standard deviation (SD), frequency, and proportions were used to describe the data. Pairwise correlation was then used to assess the association between family structure, diabetes-related clinical risk factors, and self-reported social support among grandparents. For grandchildren, pairwise correlation was conducted to assess the relationship between T2D risk factors and each CASSS subscale. Then, regression was used to further understand the relationship between social support and diabetes-related clinical risk factors, while controlling for age, sex, and race/ethnicity. Statistical significance was determined at *p*<0.05.

## RESULTS

### Grandchildren Characteristics

There were 72 grandchildren who participated in this study. Most of the participants were female (61%) and non-Hispanic white (97%) with an average age of 11.8 ± 1.5 years. The primary outcome of interest, HbA1c, was 4.9% ± 0.6. Results of additional clinical outcomes are displayed in Table 1. For social support, most grandchildren indicated that the support received from grandparents, teachers, classmates, and friends was *very important* to them: 72%, 61%, 61%, and 62%, respectively. Similarly, grandchildren reported that they *always* perceive social support from grandparents, teachers, classmates, and friends at 78%, 72%, 61%, and 62%, respectively. Subscale scores and ranges are also displayed in Table 1.

### Pairwise Correlation Between Grandchildren Social Support and Clinical Outcomes

Pairwise correlations were conducted using the total score for each subscale (see Table 2). Significant associations were found between *frequency* of social support and various clinical outcomes: grandparent support and both HbA1c (r= −0.26) and LDL (r= −0.32); classmate support and both hemoglobin A1c (r = −0.26) and BMI (r= −0.32); and friend support and hemoglobin HbA1c (r= −0.028), BMI (r= −0.24), and systolic blood pressure (r=0.29). However, there was no significant association found between teacher support and clinical outcomes. In addition, significant associations were found between *perceived importance* of social support and various clinical outcomes: grandparent support and both HbA1c (r= −0.38) and LDL (r= −0.29); teacher support and HbA1c (r= −0.30); classmate support and both HbA1c (r= −0.29) and BMI (r=0.23); and friend support and both HbA1c (r= −0.33) and systolic blood pressure (r=0.23).

### Regression Models: CASSS Subscales and Clinical Outcomes in Grandchildren

In regression models adjusting for sex, age, and race/ethnicity, we found that the frequency of receiving support from teachers was not statistically significant with clinical outcomes. However, there was a significant relationship between diastolic blood pressure and frequency of grandparent support (β= −0.34, *p*=0.04) as well as between BMI and both frequency of classmate support (β=0.56, *p*=0.038) and friend support (β=0.59, *p*=0.019). In addition, significant relationships were found between HbA1c and *perceived importance* of social support from grandparents (β= −3.99, *p*=0.005), teachers (β= −3.54, *p*=0.016), classmates (β= −2.81, *p*=0.043), and friends (β= −2.78, *p*=0.039). There were no other significant relationships between social support subscales and clinical outcomes of interest (see Table 3).

### Grandparent Characteristics

There were 66 grandparent participants in this study. Their mean age was 59.4 ± 7.4 years, and the majority were female (98.5%) and married (50.7%) or living with someone (46.1%). The primary diabetes risk factor, HbA1c, was 6.2% ± 1.4. The average social support scores among grandparents were moderately high (79 ± 3.4). All clinical outcomes are displayed in Table 1.

### Pairwise Correlation Between Grandparents’ Social Support and Clinical Outcomes

Using pairwise correlations, it was found that there was not a significant association between number of grandchildren in the home and diabetes risk factors or social support. Interestingly, there were significant associations between the number of individuals living in a household and BMI (r=0.39); triglycerides (r= −0.25); and HDL (r=0.43; see Table 4). Additional analyses, including regression models, assessing diabetes risk factors associated with grandparents raising their grandchildren in this study have been previously published.^18^

## DISCUSSION

This study examined the associations of social support and T2D risk factors among rural grandparent caregivers and their grandchildren. The grandparent caregivers in this study are representative of Appalachian Kentucky, where over 90% of the population are non-Hispanic white and where grandparent caregivers tend to be women. To our knowledge, this is one of the first studies to focus on the health of rural Appalachian grandparent caregivers and their grandchildren in a GHH. Rural Appalachian communities often have ageing or limited resources, low educational attainment, and unemployment; these inequities contribute to notable health-related challenges associated with GHH family constellations in rural communities. On the other hand, rural Appalachian communities have notable strengths, including strong family ties, collectivist values, and strong community and religious connections that may lead to favorable health outcomes.

This study brings a unique perspective by examining T2D risk in both grandparents and grandchildren of GHH. The risk factors for T2D are often shared among family members due to common patterns in lifestyle factors, physical activity, eating habits, obesity, and environmental factors. One major finding from this study is the high mean HbA1C values among the grandparents—despite grandchildren’s mean HbA1c value of 4.9% ± 0.6 (30 mmol/mol), within the normal range of ≤5.7%. Given that environmental and lifestyle factors influence T2D development, it is expedient to facilitate enjoyable physical and social activities to engage children, promote healthy lifestyle choices, and ensure continual glycemic control. In addition, culturally appropriate healthy food options should be provided at home and when eating out to promote healthy weight and reduce obesity among children.

According to the American Diabetes Association, HbA1C levels between 5.7% (39 mmol/mol) and 6.5% (48 mmol/mol) indicate prediabetes. Thus, the mean value for grandparents, 6.2% ± 1.4 (44 mmol/mol), is indicative of grandparents’ risk for T2D. Several grandparents had prediabetes (31%) or undiagnosed diabetes (28%). This high prevalence highlights the importance of prediabetes and obesity surveillance among caregiving grandparents in rural Appalachia. Individuals with prediabetes have a 4% to 19% annual risk of progression to T2D.^19^ However, prediabetes is a modifiable and preventable precursor to T2D. Understanding that rural grandparents are at disproportionate risk for T2D should lead to early intervention to mitigate disease progression from prediabetes to T2D. A comprehensive life-course approach that includes healthy eating, physical activity, and policy interventions to support both active living in safe environments and access to affordable, healthy foods is needed to mitigate prediabetes progression among rural grandparents and their grandchildren.

Provision of a healthy diet may be a challenge in rural Appalachia because of austere resources. Several counties in Appalachia have been designated rural food deserts (i.e., low-income census tract with a poverty rate ≥ 20%, where ≥ 33% of residents reside more than 10 miles from a large grocery store) by the U.S. Department of Agriculture.^17^ Access and food prices may impede adoption of healthy eating in rural communities where the cost of frequent grocery store commutes to purchase highly perishable foods, such as fresh produce, can inhibit healthy eating. Individuals with limited financial resources often purchase highly processed foods because of the longer shelf life and less risk of waste. GHH at risk for T2D and experiencing financial challenges and food insecurity should be connected to government programs such as the Supplemental Nutrition Assistance Program (SNAP)—a federally funded nutrition program to assist low-income individuals and families. Policy changes to improve local access to nutritious food and produce through community farmers markets may alleviate the access problem to fresh produce in rural communities.

In addition, prior research indicates an association between food insecurity and obesity.^20^ The mean BMI of 37.3 ± 13.0 among grandparents in this sample is indicative of obesity. This finding is consistent with previously documented high BMI among rural Appalachians.^21^ Obesity results from a combination of causes and individual factors such as behavior and genetics; other contributing factors include the food and physical activity environment, education and skills, food marketing, and community environment. Dietary patterns that are high in energy-dense, high-fat, and low-fiber foods predispose individuals to becoming overweight and obese.^22^ Overall, socioecological context and community-level factors influence obesity risk among rural Appalachians.

Furthermore, the number of people living in GHH was associated with grandparents’ triglycerides, HDL, and BMI. First, it should be noted that due to the close family structures in rural Appalachia, extended family may also live in the home, as well as other individuals with which grandparents may have a close relationship. Additional individuals living with grandfamilies is likely due to consolidation of resources in a low-socioeconomic-status environment. Thus, the relationship between the number of people living in GHH and grandparents’ triglycerides, HDL, and BMI maybe associated with food insecurity due to economic barriers, the number of individuals living within the home, and other psychosocial factors (e.g., anxiety, stress) that are associated with the development of cardiometabolic diseases. In addition, poor food choices (e.g., consuming processed foods) due to food deserts may contribute to cardiometabolic risk factors (e.g., hyperlipidemia, diabetes, obesity) experienced by rural Appalachians. Rural residents living in economically disadvantaged areas may experience the greatest risk for excess body weight.

Average social support scores among grandparents suggested moderately high perceived social support (79 ± 3.4). This is not surprising, because rural Appalachian residents have a high sense of social bonds, social relations, and community ties. Social support is an aspect of human social relationship by which emotional, instrumental, or financial help can be obtained from an individual’s social network.^23^ According to Sherbourne and Stewart,^12^ there are 5 dimensions of social support that can be evaluated via the MOS-SSS: (1) emotional support (i.e., the expression of positive affect, empathetic understanding, and the encouragement of expressions of feelings); (2) informational support (i.e., the offering of advice, information, guidance or feedback); (3) tangible support (i.e., the provision of material aid or behavioral assistance); (4) positive social interaction (i.e., the availability of other persons to do fun things with you); and (5) affectionate support (expressions of love and affection). It appears that grandparents draw from these different sources of social support. Research indicates social support from family and significant others that promote healthy behaviors are associated with better T2D self-care.^24^ By extension, greater social support may improve self-care to prevent disease progression from prediabetes to T2D.

For these reasons, engaging social support to improve self-care behavior can be important in mitigating T2D risk among grandparents. Grandparents and grandchild(ren) relationships constitute a crucial element of the social support network for older adults. Grandparenthood is an indication of social bonds, which may act as a buffer against the negative social and psychological consequences of aging.^25^ However, in the era of COVID-19 pandemic, grandparents may not be able to benefit from close relationships outside the home due to social distancing recommended to minimize risk of exposure.

For grandchildren, social support provided by grandparents had no association with T2D risk factors, whereas support from teachers, classmates, and close friends was associated with HbA1C, BMI, and blood pressure. It is estimated that children and adolescents spend approximately 7 hours of their daytime engaged in academic and leisure activities with peers and close friends, and these relationships provide a critical source of emotional support.^26^ These findings support the use of peer support and school settings as mechanisms for interventions to reduce T2D in adolescents, particularly those from GHH. Consequently, it is critical for clinicians and researchers to understand the ways that relationships—including those with classmates, close friends, and teachers—affect the ability of grandchildren from GHH to adapt and adhere to healthy lifestyles. However, in the era of COVID-19 pandemic, social support for grandchildren may have been significantly impacted due to school closures and remote learning methods. During this and future disease outbreaks, many kids may not be able to visit their friends and peers due to recommended physical restrictions, including social distancing, quarantine, and isolation to prevent disease spread. Innovative methods using technological connections, such as video calls, may reinforce social support during periods of social isolation.

Despite the strengths of this study, some limitations should be acknowledged. First, the sample size was small, and results are not generalizable to *all* grandparents who are caring for their grandchildren or even to all Appalachian grandparents. Second, because the study collected cross-sectional data, it cannot determine causal relationships between grandparents’ health status and caregiving responsibility. Third, information on grandchildren’s peers and the type of support received was not collected, nor social support subscales stratified because of the small sample size. Lastly, to our knowledge, this is the only study that has attempted to look at T2D risk in GHH in Appalachia. It cannot determine whether T2D risk is the same, worse, or better in GHH compared to traditional family structures. However, future studies should (1) collect longitudinal data to better elucidate the effect of caregiving on grandparents; (2) conduct qualitative interviews with grandchildren to better understand the nuances of perceived social support from various sources; (3) develop interventions to leverage established community organizations (e.g., faith-based organizations, schools, and community centers); (4) reduce T2D risk through programs focused on physical activity, healthy eating, as well as stress and anxiety reduction; and (5) investigate whether T2D risk factors among GHH are significantly different from those among traditional family structures in Appalachia.

## IMPLICATIONS

Screening and early detection are critical to slow progression of prediabetes to T2D. Understanding the prevalence of prediabetes and T2D risk among GHH will allow tailored interventions to include important information and appropriate activities that might prevent the progression to T2D. Healthcare providers and researchers should take advantage of the high perceived social support among grandparents by providing GHH and their family members with diabetes prevention information and encourage lifestyle changes at the family and socioecological level. For grandparents, increased responsibility may provide a determinant of health due to the potential stress of caregiving for grandchildren. Future research should assess how this caregiver role may affect T2D risk factors or T2D self-care practices in grandparents. In addition, these findings suggest the use of social support in school settings as mechanisms for interventions could be useful to reduce risk of T2D for grandchildren. A larger sample is needed to perform additional analysis and provide clarity on statistically significant associations.

**SUMMARY BOX**

**What is already known about this topic?**

There is not much known about perceived social support and T2D risk factors among grandparent-headed households in Appalachian Kentucky.

**What is added by this report?**

The findings suggest that assessing risk factors for grandparents and grandchildren living in grandparent-headed households is important to mitigating T2D risk throughout the lifespan.

**What are the implications for future research?**

Additional research is needed to evaluate long-term risk for grandparent and children of this family unit. Similarly, research assessing how social support can be leveraged for the family unit as well as for grandparents and grandchildren, independently, is needed.

## Figures and Tables

**Table 1:** Sample Characteristics for Grandparents and Grandchildren

Characteristic	Grandparents (n=66)	Grandchildren (n=72)
**Mean Age** (years)	59.4 ± 7.4	11.8 ± 1.5
**Gender**		
Male	1 (1.5%)	28 (38.8%)
Female	65 (98.5%)	44 (44%)
**Marital Status**		
Single	2 (3.1%)	—
Married	33 (50.7%)	—
Living with someone	30 (46.1%)	—
**HbA1c** (%)	6.2 ± 1.4	4.9 ± 0.6
**Cholesterol** (mg/dL)		
LDL cholesterol	98.8 ± 48.1	75.02 ± 25.3
HDL cholesterol	50.0 ± 35.5	44 ± 13.5
Triglycerides	219.0 ± 137.6	125.8 ± 82.6
Total Cholesterol	185.8 (± 5.9)	144.7 ± 29.0
**BMI**	37.3 ± 13.0	24.49 ± 8.0
**Systolic Blood Pressure** (mmHg)	90.8 ± 100.5	114 ± 12.9
**Diastolic Blood Pressure** (mmHg)	131.5 ± 20.8	68.6 ± 12.2
**MOS Social Support**	79.2 ± 27.6	—
**CASSS Frequency of Grandparent Support**	—	61.0 ± 11.0
**CASSS Frequency of Teacher Support**	—	59.3 ± 10.5
**CASSS Frequency of Classmate Support**	—	54.4 ± 12.2
**CASSS Frequency of Friend Support**	—	68.6 ± 12.2
**CASSS Perceived Importance of Grandparent Support**	—	29.8 ± 5.7
**CASSS Perceived Importance of Teacher Support**	—	27.7 ± 5.6
**CASSS Perceived Importance of Classmate Support**	—	26.4± 5.3
**CASSS Perceived Importance of Friend Support**	—	26.9 ± 5.3

**Table 2:** Correlation between Grandchildren CASSS Responses and Type 2 Diabetes Risk Factors

	Frequency of Social Support				Perceived Importance of Social Support			
	Grandparent	Teacher	Classmate	Friend	Grandparent	Teacher	Classmate	Friend
**HbA1c**	−0.26*	−0.20	−0.25*	−0.28*	−0.38*	−0.30*	−0.29*	−0.33*
**BMI**	−0.09	0.12	0.32*	0.24*	0.06	0.03	0.23*	0.15
**LDL**	−0.32*	−0.21	−0.14	−0.18	−0.29*	0.01	−0.17	−0.14
**HDL**	0.03	0.07	0.18	0.19	0.08	0.09	0.03	0.13
**Triglycerides**	0.02	0.04	0.00	−0.06	−0.02	−0.12	−0.08	−0.10
**Total Cholesterol**	−0.09	−0.01	−0.04	−0.07	−0.14	0.03	−0.17	−0.08
**Systolic Blood Pressure**	−0.04	0.10	0.21	0.29*	0.10	0.21	0.16	0.23*
**Diastolic Blood Pressure**	−0.14	−0.02	0.01	0.14	0.02	0.14	−0.02	0.13

NOTES:

* Indicates statistical significance at *p*<0.05.

**Table 3:** Adjusted Regression Between Grandchildren’s Social Support and Clinical Outcomes

	Frequency of Social Support β (*p*-value)				Perceived Importance of Social Support β (*p*-value)			
	Grand-parent	Teacher	Classmate	Friend	Grand-parent	Teacher	Classmate	Friend
**HbA1c**	−3.73 (0.19)	−2.50 (0.37)	−2.77 (0.40)	−3.95 (0.19)	−3.99 (0.00*)	−3.5 (0.01*)	−2.81 (0.04*)	−2.78 (0.03*)
**BMI**	0.12 (0.58)	0.27 (0.22)	0.55 (0.03*)	0.59 (0.02*)	0.05 (0.66)	0.11 (0.33)	0.16 (0.16)	0.13 (0.23)
**LDL**	−0.18 (0.21)	−0.07 (0.60)	−0.02 (0.88)	−0.00 (0.98)	0.01 (0.94)	0.07 (0.31)	0.06 (0.37)	0.02 (0.78)
**HDL**	−0.09 (0.65)	0.03 (0.88)	0.17 (0.44)	0.27 (0.18)	0.06 (0.50)	0.09 (0.37)	0.06 (0.46)	0.11 (0.21)
**Triglycerides**	−0.54 (0.87)	−0.35 (0.91)	−0.42 (0.91)	0.73 (0.84)	1.70 (0.30)	1.19 (0.49)	1.05 (0.52)	0.98 (0.53)
**Total Cholesterol**	0.08 (0.50)	0.03 (0.81)	−0.00 (0.99)	0.01 (0.94)	−0.02 (0.74)	−0.01 (0.84)	−0.06 (0.33)	0.00 (0.99)
**Diastolic Blood Pressure**	−0.34 (0.04*)	−0.30 (0.08)	−0.29 (0.12)	−0.24 (0.17)	−0.07 (0.40)	−0.05 (0.57)	−0.143 (0.08)	−0.10 (0.21)
**Systolic Blood Pressure**	0.13 (0.37)	0.23 (0.11)	0.27 (0.11)	0.09 (0.15)	0.06 (0.41)	0.08 (0.29)	0.11 (0.14)	0.09 (0.15)

NOTES:

* Indicates statistical significance at *p*<0.05; adjusted for sex, age, and race/ethnicity.

**Table 4:** Correlation between Grandparents Characteristics and Type 2 Diabetes Risk Factors

	No. of Grandchildren in the Home	No. of Individuals Living in Household
**Total Cholesterol**	−0.09	−0.14
**Triglycerides**	−0.15	−0.25*
**LDL**	0.02	−0.13
**HDL**	0.22	0.43*
**HbA1c**	0.17	0.09
**Systolic Blood Pressure**	−0.06	−0.04
**Diastolic Blood Pressure**	0.06	−0.10
**BMI**	−0.13	0.39*
**Social Support**	0.08	0.07

NOTES:

* Indicates statistical significance at *p*<0.05.
